# A bio-logging dataset on the diving behaviours of juvenile sea turtles from the southwestern Indian Ocean

**DOI:** 10.1038/s41597-026-06793-4

**Published:** 2026-06-10

**Authors:** Mohan Julien, Pierre Gogendeau, Alexandre Boyer, Andrea Goharzadeh, Titouan Etienne, Cheryl Sanchez, Luke A’Bear, Serge Bernard, Sylvain Bonhommeau

**Affiliations:** 1 IFREMER, Department of Biological Resources and Environment (RBE), Indian Ocean Delegation (DOI), Le Port, Réunion; 2 CNRS, University of Montpellier, Laboratory of Computer Science, Robotics, and Microelectronics (LIRMM), Montpellier, France; 3 SIF, Seychelles Islands Foundation, Victoria, Seychelles

**Keywords:** Animal behaviour, Sensors and probes

## Abstract

In recent decades, international and national regulations have contributed to slowing population declines and promoting the stabilization and recovery of marine turtle populations. Numerous studies have highlighted the intricate interconnections between sea turtles, particularly juveniles, and their surrounding ecosystems, underscoring the critical importance of long-term monitoring in marine conservation strategies. Aligned with conservation efforts in the region, this work provides a high-resolution dataset on the diving behavior of juvenile green and hawksbill turtles from four sites: Reunion Island, Mayotte, Europa Island, and Aldabra Atoll. Between 2021 and 2022, we collected over 100,000 records from 37 juveniles using open-source bio-loggers using LoRaWAN technology. The dataset, openly available on SEANOE, includes raw and processed data following FAIR principles. We believe that it offers a supporting resource for studying juvenile sea turtles and demonstrates the potential of open-source technologies for biodiversity monitoring.

## Background & Summary

While sea turtles have often showed long-term declines in abundance and many populations are listed as Vulnerable, Endangered or Critically, conservation efforts have led to recoveries across many species and populations^1^. The French National Action Plan (NAP) for the Conservation of Sea Turtles in the French Southwest Indian Ocean Territories highlights the need to reduce marine turtle mortality, protect critical habitats, and improve scientific knowledge, particularly regarding the vulnerable juvenile stage^2,3^. It also emphasizes the importance of comparing data from both pristine areas and zones under anthropic pressure to better understand the impacts of human activities. Juvenile turtles are known for their ecological plasticity and variable foraging behaviour across environments and populations^4–9^. Understanding this variability is essential for conservation planning. Tagging studies have proven effective in uncovering spatial distribution, habitat use, and behavioural rhythms, such as diel and seasonal patterns^10–12^. Long-term datasets combining temperature, depth, dive time, and surface intervals provide a valuable foundation for modelling breathing cycles, diving strategies, and responses to environmental conditions^13–17^.

This work relies on a fully customizable, cost-efficient open-source bio-logger and associated receiving stations based on LoRaWAN technology (Long Range Wide Area Network). The bio-loggers are designed to record near-real-time information on turtle dive and surface events, including depth profiles, durations, and temperature. In this study, GPS positioning and movement tracking were not part of the final configuration, but the core electronics include a GPS module, an accelerometer-gyroscope, and a light sensor that can be activated if required. LoRaWAN is a low-power wireless protocol capable of long-distance transmission and provides a complementary alternative to Argos, Iridium or GSM/LTE technologies for coastal species^18–21^. It is designed for efficient and robust time-series transmission from heterogeneous sensors, but requires nearby gateways for data reception. Existing approaches using Argos or GSM have already shown successful results where Argos systems can rely on coastal ground stations to increase bandwidth and link reliability, and GSM tags exploit existing mobile networks with good results. LoRaWAN, by contrast, is far more energy-efficient, with per-message consumption typically 20-100 × lower than GSM and even more compared to Argos. While GSM supports infrequent but large uploads, LoRaWAN enables frequent small packets. In this work, we also demonstrate that autonomous LoRaWAN receiving stations can be deployed and maintained in remote areas, even by non-specialists. The hardware and network operation are detailed in the Methods section below. This system has enabled the deployment of four bio-logger networks for the purpose of studying the habitats and behaviour of 37 juvenile green (*Chelonia mydas*) and hawksbill (*Eretmochelys imbricata*) turtles at four key sites, namely the Reunion Island, Mayotte, the Europa Island, and the Aldabra Atoll, all in the SWOI region.

This field work yielded a high-resolution dataset^22^ that offers detailed insights into their underwater activities. The dataset, after cleaning and prepossessing stages, comprises 100,875 records of temperature, dive profile and duration of surface events from 34 juvenile individuals. The median number of messages received per tag is 1,371. The median transmission period is 40.5 days and the average message frequency per tag is close to one message per hour. The original data are enriched with additional explanatory variables derived from the raw dive profiles (dive variability, dive rate of change, number of “activity phases”) and with categorical variables to facilitate categorisation by time of day and season and to facilitate filtering by type of dive/surface events (i.e. “very-shallow” up to “very-deep” for depths or “very-short” up to “very-long” for durations). A repository with the complete dataset^22^, associated processing scripts, and illustrative reports is publicly available on SEANOE (SEA scieNtific Open data Edition), an open scientific data repository in the field of marine sciences.

It is anticipated that this data will support ongoing efforts to model habitat use, foraging strategies and responses to environmental change, thereby contributing to a better understanding and conservation of these threatened species. This dataset offers a relatively high frequency of observations, which is comparable to the methods used in the reference studies mentioned in the *Technical Validation* section. In addition, the data are interoperable with existing datasets produced with Argos tags, such as the SPOT or SPLASH series produced by Wildlife Computers. Furthermore, the data comprises underwater temperature measurements that can be examined separately from turtle studies, offering possibilities for broader applications in various environmental or ecological scenarios.

## Methods

### Areas and species studied

**Fig. 1:**
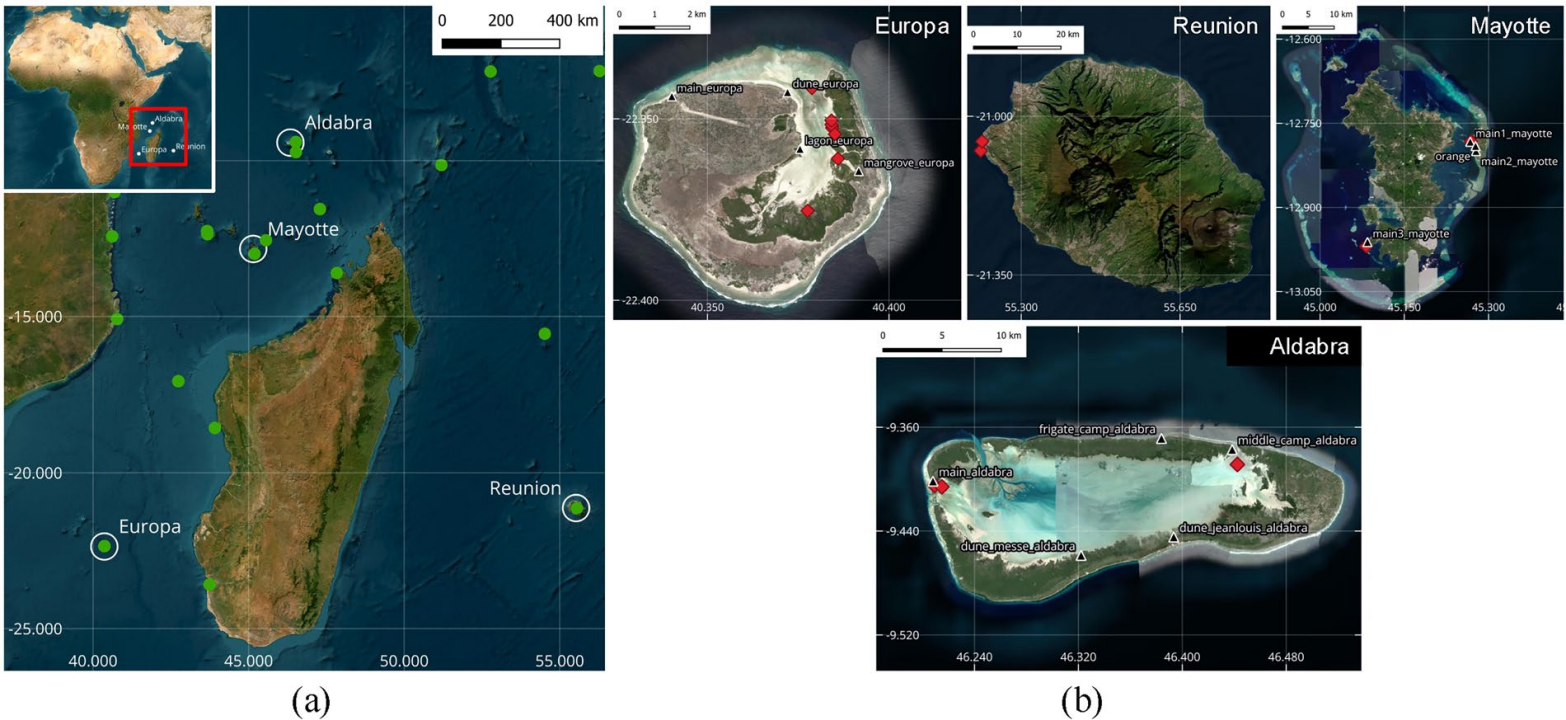
(**a**) The four study sites of the IOT project are of the major nesting areas of the SWOI. Large white circles mark the site locations were the data has been collected. Small green dots are reported nesting sites of green turtles^45^. (**b**) Satellite maps of the four sites. Red diamonds are turtle capture locations, black triangles correspond to locations of the LoRa stations deployed on Europa Island and Aldabra Atoll for the IOT project.

The study was conducted across four sites in the western Indian Ocean, encompassing the French territories of the Reunion Island, Mayotte, the Europa Island, and the Aldabra Atoll in the Seychelles (Fig. 1). These regions are crucial habitats for juvenile marine turtles and encompass a diverse array of environmental conditions. The Reunion Island, to the east of Madagascar, features a wide variety of marine habitats, including coral reefs, rocky shores, and deep-water areas. Mayotte, to the north of the Mozambique Channel, is distinguished by its extensive lagoon system and well-preserved coral reefs. These habitats offer refuge and abundant food resources for juvenile sea turtles. Europa, a 30 km^2^ isolated island in the Mozambique Channel, presents a unique environment for studying marine turtle behaviour in a low antrogenic impact site. The island pristine waters, preserved mangroves and relatively undisturbed ecosystem are recognized as important nesting sites, especially for green turtles. The Aldabra Atoll, designated as a UNESCO World Heritage Site and recognized as one of the largest coral atolls worldwide, serves as a significant sanctuary for marine life. Its shallow lagoons, mangroves, and seagrass beds provide essential habitats for juvenile turtles.

The study focused on juvenile green turtles and hawksbill turtles. Green turtles are widely distributed across tropical and subtropical seas, and they are the most abundant species in the western Indian Ocean, where all life stages occur^3^. Their diet transitions from omnivorous with a carnivorous preference in the juvenile stage, where they consume invertebrates and fish eggs, to primarily herbivorous in adulthood, feeding on algae and seagrasses^23^. During the sub-adult stage, their diet becomes exclusively herbivorous, a pattern that continues throughout their life^24^. Juvenile green turtles have been observed in cooler waters off South Africa, favouring coral reef and mangrove habitats that provide protection and food resources, mainly marine seagrasses and red algae^25^. Genetic studies have identified three green turtle subpopulations in the region: one in the southern Mozambique Channel, closely related to the Atlantic stock, another in the northern channel, and one near the Seychelles^26^. Hawksbill turtles are primarily distributed around the equator and reproduce during the warm season on remote beaches and islands. Following an initial pelagic phase, juveniles (20-25 cm in size) migrate to coral reefs, which serve as feeding and growth habitats. Hawksbill turtles are generally omnivorous, consuming ascidians, sponges, crustaceans, mollusks, sea urchins, fish, echinoderms, and marine algae, with sponges being the predominant component of their diet^27–29^. Although suitable coral reef habitats extend into the southwestern Indian Ocean, little is known about this species abundance in the region. To date, genetic research on hawksbill turtles has been limited but an ongoing project (TIMOI) aims at providing new information on the population structure of this species in the South-West Indian Ocean.

### New cost-efficient open-source sea turtle tags

**Fig. 2:**
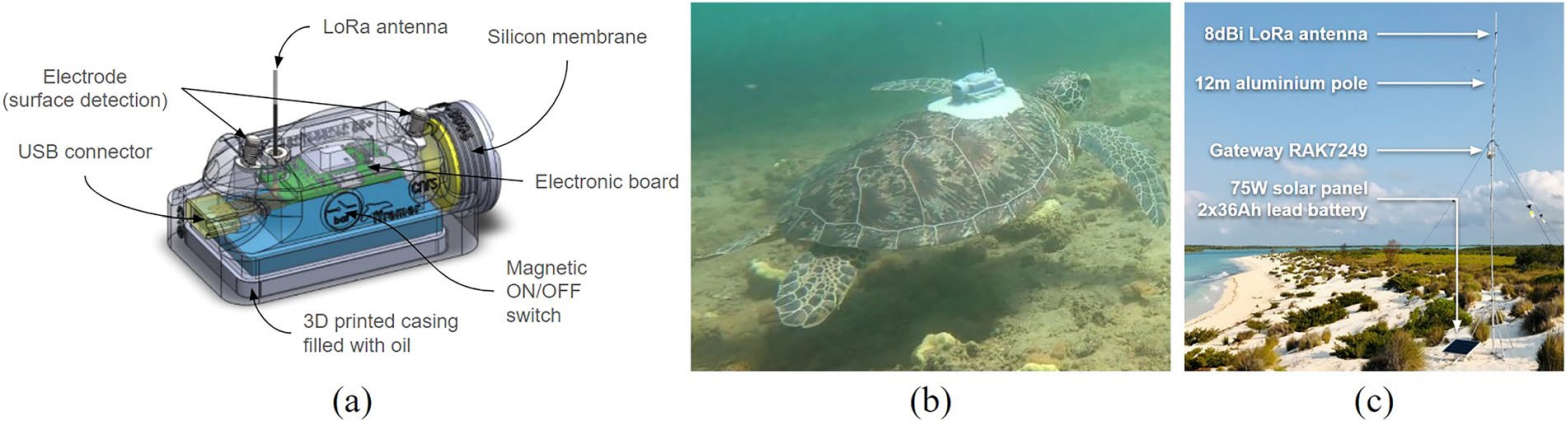
(**a**) Annotated 3D rendering of the Indian Ocean sea Turtle tags. Dimension: 9 × 4.5 × 4 cm. Weight: 150 g in air and 26 g in salt water. (**b**) A juvenile green turtle tagged in Mayotte in July 2021 with the IOT tag. (**c**) Picture of the Ifremer’s autonomous solar-powered LoRa station deployed on Europa Island.

The recording of dive and surface events have been done through electronic tags designed by the partners of the IOT project and specifically tailored for marine turtles (Fig. 2a,b). In contrast to the majority of sea turtle tags, which depend on satellite communication, the data transmission in this case relies on the LoRa/LoRaWAN technology. The low energy consumption and capacity for transmission over long distances (up to several dozen kilometers under optimal conditions) render it an appealing solution for the design of miniature and long-lasting devices. The tags transmit data in near real-time, with the last measurement transmitted each time the individual reaches the surface. Each tag can be built for approximately 100 – 200, and the fully open-source design enables users to assemble and adapt the system as a DIY solution.

The messages sent by the tags are collected using a network of LoRa gateways located within a few kilometers of the tagged turtle home ranges. The receiving gateways relay the messages to a centralised time-series online database, which can be further queried to download the tags data. Due to the specificity of the different sites in terms of internet access and power supply, we developed a versatile infrastructure with hardware and data flow that make use of: i) a commercial LoRa infrastructure which maximises the coverage for urbanised coastal sites like in the Reunion Island and Mayotte. ii) a private LoRa network dedicated to the remote sites that is based on autonomous solar-powered gateways (Fig. 2c). Gateways on the Aldabra Atoll and Europa Island have been installed by IOT partners. The gateways location on these two sites are reported in Fig. 1b. The gateways on Europa Island are separated by a few kilometers (min 1.8 km, max 5.7 km) and distributed around the internal lagoon and off the reef on the north of the island. The gateways on the Aldabra Atoll are distributed around the inner lagoon and are separated by distances ranging from 8.9 km to 25.4 km. Exact coordinates and associated information are available in the public repository in CSV format.

The transmitted data comprises a number identifying the measurement, a timestamp, the last dive profile, the last duration spent on the surface, the average temperature recorded during this dive, and the LoRaWAN metadata. The raw data of the two embedded pressure and temperature sensors (MS5803-01BA, MS5803-30BA) are used to record the individual dive profile with an accuracy of 10 cm and a maximum depth of 25.5 m. To maintain depth measurements within the 10 cm accuracy range and account for sensor drift over time or changes in atmospheric pressure, we implemented a minimum sliding filter over a 2-hour window to continuously establish a reference for pressure checks and depth calculations. The dive profile is compressed into 20 values with adaptive time steps to accommodate longer dive times. The minimum time step is 15 seconds, with a doubling of its value occurring when the dive duration exceeds the value of 20 times the time step. For example, if the duration of the dive does not exceed 5min (20 × 15s), the profile time step will be equal to 15s. As soon as the dive lasts longer than 5min, the time step is redefined to 30s. This value will be doubled again (60s) if the duration finally exceeds 10 min (20 × 30s), and so on. A surface detection mechanism has been integrated into the system to record surface events. The detection is based on the combination of a conductivity measurement, utilising the embedded Analogue to Digital Converter (ADC) to capture the signal between two electrodes positioned on the casing and of a pressure measurement from the MS5803-01BA sensors (Operating range: 10 to 1300 mbar). The casing is a custom design created using 3D printing technology and manufactured in Tough 1500 Formlabs resin. To withstand underwater pressure, the housing is vacuumed and then filled with non-conductive oil. A silicon membrane at the back is employed to ensure the transmission of external water pressure to the internal sensors. The unit has been tested at depths exceeding 150 metres. The resolution of the temperature sensors themselves is reported to be less than 0.01°C, but thermal noise caused by the heat of the electronic board during operation reduces the actual accuracy. In addition, rapid temperature changes of less than a few minutes cannot be properly recorded due to the thermal inertia of the tags, as the sensors are immersed in oil. We tried to minimise the size and weight of the tag in order to ensure that it would be as unobtrusive as possible for the tagged animals. The deployed tag version weighs approximately 150 g in air (26 g in salt water) and measures 9 × 4.5 × 4 centimetres.

Reliance on LoRaWAN transmission can introduce a spatial bias, as coverage is limited to the lagoons under study. As a result, no data are received when individuals move beyond the gateway’s transmission range. However, juvenile turtles are known to occupy relatively small home ranges^30–32^, and the distribution of message arrival times in this study suggests that individuals generally remain within coverage or leave it only briefly. The average message frequency per tag across all sites is approximately one message per hour (1.03), with very few gaps longer than 24 hours, indicating that the turtles are typically within transmission range.

### Capture and tagging protocols

The tagging process followed a standardised protocol reviewed by an ethical committee and local regulations, with all personnel involved receiving comprehensive training. In accordance with the provisions of the Rural and Maritime Fishing Code (Articles R.214-87 to R.214-126) and the 3Rs principle for EU Member States (Article 4 of Directive 2010/63/EU), we obtained authorization from the Ministry of Higher Education, Research and Innovation to carry out the capture and tagging of marine turtles (APAFIS#26501-2019111414383771 v2) and by the National Council for Nature Protection (2020-00664-031-001). In addition, for each territory, specific permits are also required for the capture, handling, and tagging of protected species. The authorization to tag sea turtles in the Marine Reserve of Reunion island has been approved by the Prefet de la Reunion (DECISION N°2020-45 and DECISION DEAL/SEB/UBIO/2022-08). The authorization for tagging in Mayotte has been approved (arrêté préfectoral 2020/DEAL/SEPR/493 and 2021/DEAL/SEPR/161). The authorization in Europa has been approved by the Prefet des Terres Australes et Antarctiques Françaises (arrêté 2020-60). The authorization for tagging in Aldabra has been approved by the Seychelles Islands Foundation. The authorization for the use of LoRa and VSAT radiofrequencies in Aldabra has been approved by the Seychelles Bureau of Standards.

To meet ethical and permitting criteria and ensure the success of tagging experiments, tags must have a limited impact on animal behaviour. A substantial body of literature documents such effects, indicating that individuals may return to normal activity within a few days, depending on the level of human disturbance in their environment^33–35^. We followed general guidelines by ensuring that tag weight remained below 3% of the gravitational force acting on the animal’s body, as recommended by recent research^36^. Our tags weigh 150 g in air and 26 g in salt water, which complies with this recommendation and is expected to limit disturbance in individuals weighing more than 5 kg. The weight and size are comparable to those of widely used commercial Argos tags, such as the Wildlife Computers SPLASH10 and the Lotek Wireless F6G series.

Training sessions were held at the *Kelonia Care Centre* on Reunion Island, where the staff were instructed on turtle capture techniques and tag attachment methods. Additionally, the team was trained in the TORSOOI identification protocol.

Turtle capture was performed using various methods, including on-foot pursuit, snorkelling or boat-based capture, commonly known as the rodeo technique^37^. After capture, detailed morphological measurements, such as weight, straight shell length, and curved shell length, were systematically recorded. Prior to tag fixation, the designated shell area is cleaned and dried in order to ensure optimal adhesion. A resin with a low exothermic reaction (epoxy POX150-PRO) is then applied in a thin layer to the central region of the shell and partially extended to adjacent areas. The resin fully polymerises in approximately two hours, with any irregularities smoothed during the process to minimise fouling from biological growth or algae. During the drying process, the turtles are protected from the sun and their eyes covered with a wet towel to limit their stress. Once the resin has fully hardened, the turtle is released near its original capture location in order to reduce environmental disruption.

The experiments started in May 2021 on Europa Island and were concluded in January 2022 on Reunion Island. A total of 37 individuals were tagged, 9 on the Reunion Island, 10 on Mayotte, 10 on the Aldabra Atoll and 8 on the Europa Island. Of the 37 individuals, 29 were green turtles and 8 were hawksbill turtles. Table 1 gives the detailed list of tagged turtles with corresponding tag names, complete capture and release information and morphological measurements. This table in CSV format has been included in the public repository.

**Table 1:** Detailed list of tagged turtles with corresponding tag names, capture information and morphological measurements.

Name	Location	Capture time	Capture coord. (lat, lon)	Species	Weight (kg)	Curved length (cm)	Curved width (cm)	Straight length (cm)
IOT-014	Europa	2021-05-25	−22.375333, 40.377575	Green	20.6	56.0	—	52.0
IOT-021	Europa	2021-06-01	−22.360980, 40.385940	Green	15.2	52.0	—	49.0
IOT-Sun	Europa	2021-06-02	−22.352000, 40.384440	Green	18.8	57.0	—	54.0
IOT-015	Europa	2021-06-07	−22.352500, 40.384330	Green	19.1	58.0	—	54.0
IOT-018	Europa	2021-06-07	−22.354210, 40.385100	Green	24.2	62.0	—	57.0
IOT-017	Europa	2021-06-08	−22.351580, 40.384140	Green	13.3	54.5	—	51.0
IOT-010	Europa	2021-06-08	−22.350500, 40.384160	Green	35.0	68.5	—	63.1
IOT-016	Europa	2021-06-11	−22.341510, 40.378800	Hawks.	11.8	50.0	—	48.0
IOT-009	Mayotte	2021-07-19	−12.782344, 45.268829	Green	17.3	54.6	45.6	—
IOT-013	Mayotte	2021-07-19	−12.782344, 45.268829	Hawks.	7.1	41.2	35.0	—
IOT-011	Mayotte	2021-07-20	−2.782344, 45.268829	Green	12.6	48.9	45.9	—
IOT-006	Mayotte	2021-07-20	−12.782344, 45.268829	Hawks.	11.0	47.5	41.5	—
IOT-023	Mayotte	2021-07-22	−12.782344, 45.268829	Hawks.	6.3	39.3	36.1	—
IOT-022	Mayotte	2021-07-22	−12.782344, 45.268829	Green	10.0	43.8	41.0	—
IOT-025	Mayotte	2021-08-30	−12.969139, 45.082130	Green	28.5	64.5	57.5	—
IOT-026	Mayotte	2021-08-30	−12.969139, 45.082130	Green	37.0	69.7	62.2	—
IOT-027	Mayotte	2021-08-30	−12.969139, 45.082130	Green	20.2	55.9	51.0	—
IOT-028	Mayotte	2021-08-30	−2.969139, 45.082130	Green	14.7	51.6	43.6	—
IOT-036	Aldabra	2021-10-15	−9.404900, 46.209326	Green	—	64.8	61.0	60.0
IOT-030	Aldabra	2021-10-28	−9.388736, 46.442443	Green	7.8	41.7	30.4	38.6
IOT-037	Aldabra	2021-10-28	−9.388736, 46.442443	Green	—	44.4	40.0	40.8
IOT-029	Aldabra	2021-10-29	−9.388736, 46.442443	Hawks.	11.9	51.2	43.2	47.2
IOT-035	Aldabra	2021-10-29	−9.388736, 46.442443	Green	9.8	44.1	42.6	41.8
IOT-032	Aldabra	2021-11-25	−9.404900, 46.209326	Hawks.	14.8	53.4	48.0	49.3
IOT-039	Aldabra	2021-11-25	−9.404900, 46.209326	Hawks.	26.2	63.8	56.1	59.8
IOT-034	Aldabra	2021-12-29	−9.405905, 46.214882	Green	10.0	43.8	42.9	42.6
IOT-038	Aldabra	2021-12-29	−9.405905, 46.214882	Green	17.5	53.6	48.8	51.0
IOT-033	Aldabra	2022-04-07	−9.405905, 46.214882	Green	20.0	54.0	48.5	51.0
IOT-042	Reunion	2021-12-16	−21.075947, 55.210483	Green	52.0	78.0	74.0	72.0
IOT-043	Reunion	2021-12-16	−21.075947, 55.210483	Green	17.1	53.7	52.5	50.0
IOT-046	Reunion	2022-01-06	−21.075947, 55.210483	Green	21.5	56.7	51.6	53.0
IOT-047	Reunion	2022-01-06	−21.075947, 55.210483	Green	10.3	45.5	41.0	42.5
IOT-048	Reunion	2022-01-06	−21.055616, 55.213740	Green	10.4	43.7	41.0	40.5
IOT-049	Reunion	2022-01-06	−21.055616, 55.213740	Green	24.5	62.0	56.0	56.0
IOT-041	Reunion	2022-01-07	−21.055616, 55.213740	Hawks.	24.5	65.0	56.2	—
IOT-044	Reunion	2022-01-07	−21.055616, 55.213740	Green	13.0	48.0	42.7	—
IOT-045	Reunion	2022-01-07	−21.055616, 55.213740	Green	35.0	67.5	58.4	—

### Data post-processing

**Fig. 3:**
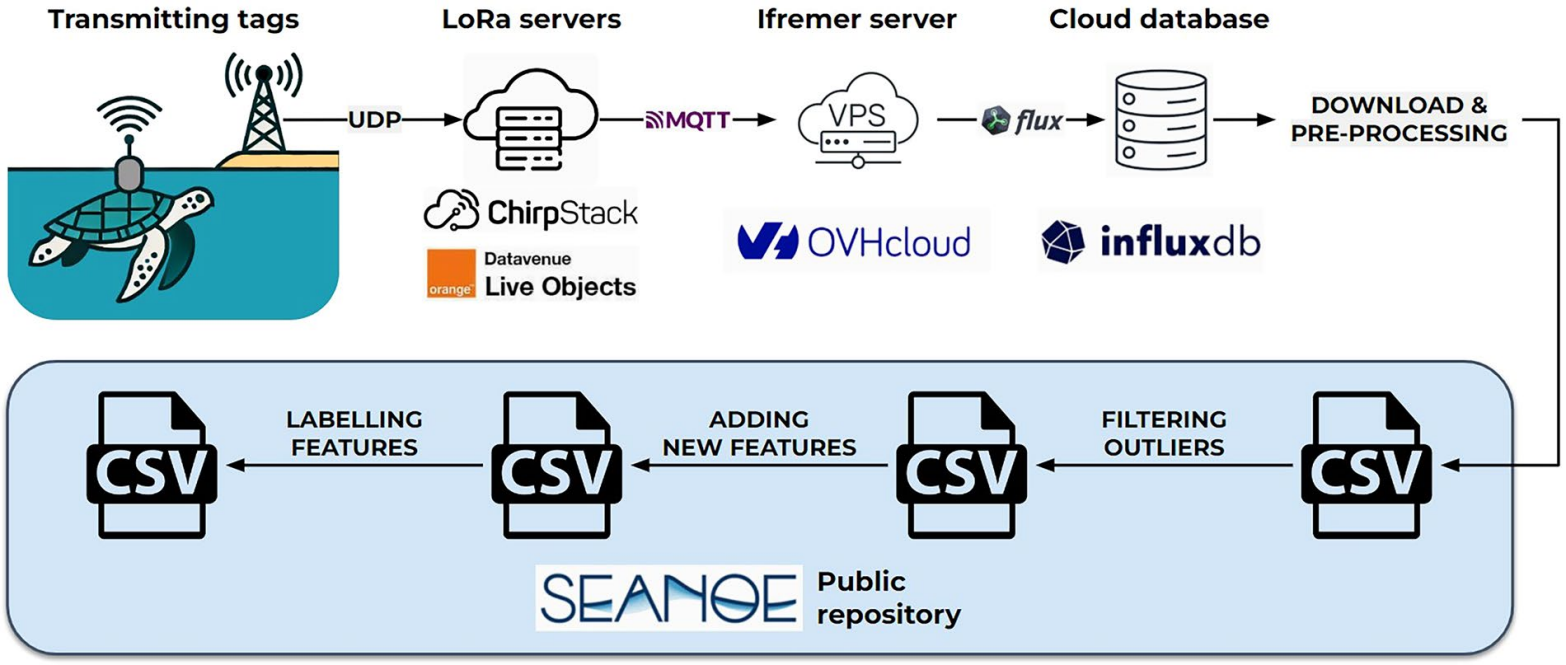
Overview of the entire data flow, from the data transmission of tags attached to turtles, through data collection and processing, to the final dataset versions available on the SEANOE platform.

The complete data flow is illustrated in Fig. 3. The original raw messages sent by the tags and collected through the LoRa gateways are stored in a private database hosted on the Ifremer server and is not publicly available. Data in its rawest form made available in the public repository has been produced by: 1) Downloading the raw data from the database. 2) Curating the data to remove entries with corrupted messages due to very low battery levels or premature failures. 3) Formatting the data by adding a new column with the correspondence between tags IDs and names, renaming some others columns and suppressing irrelevant ones (i.e. those associated with the unused GPS and the DB query). The corresponding file is named iot_all_turtles_preproc.csv. In addition, we provide three processed versions of the dataset, one with outliers filtered, one with additional explanatory variables derived from the initial measurements and one with supplementary categorical variables. The script that generates these versions, named data_analysis_1.py, have been included in the public repository or can be downloaded from the dedicated GitHub project (see section Code Availability). The complete description of content, available analysis and associated output files is given in section Data Records.

#### Filtering the data

In this dataset, outliers in the measurements may be attributed to several potential causes. These include inaccurate surface detection, likely due to misidentification caused by sensor interference or “fooling” effects. Additionally, extended surface intervals could result from environmental factors such as low tide, where the water level remains close to the top of the turtle’s shell but beneath the tag, causing erroneous data readings. Other undetermined technical issues or sensor malfunctions could also contribute to these anomalies. The filtering function removes outliers from the surface duration (tsurf_s) and dive duration (tdive_s) columns based on an interquartile range (IQR) approach. The function calculates the first (Q1) and third (Q3) quantiles of the data at the 5th and 95th percentiles, respectively. The IQR is computed as the difference between Q3 and Q1 as in equation (1) The outlier boundaries are determined by extending 1.5 times the IQR below Q1 and above Q3 as in equations (2) and (3). Data points that fall outside the lower or upper bounds are classified as outliers.

1 IQR=Q3−Q1

2 $$\,{\rm{lower\; bound}}\,=Q1-1.5\times IQR$$

3 $$\,{\rm{upper\; bound}}\,=Q3+1.5\times IQR$$

#### Adding new features

During these steps we compute new features (variables) based on the measurement of the surface duration (tsurf_s), dive duration (tdive_s) and raw dive profile (profile_m). We first analyse the message sequences for each tag to retrieve, when applicable, the surface duration before and after the current dive event (tsurf_predive_s and tsurf_postdive_s). Then we compute the ratio between surface and dive duration (divetosurf_pre_ratio and divetosurf_post_ratio) to highlight the relationship between these two features and facilitate further analyses by users. The message sent by the tags contains only the surface duration before the current dive (tsurf_s). Thus, if the next message has been lost (not received) the post-diving surface duration and associated ratio are not available. To help characterise the type of dives recorded, we processed three new features from the raw dive profile: its variability, the average rate of change and the count of “activity phases”. As mentioned before, the raw dive profile is an array of *n* = 20 depth values recorded during the turtle’s underwater trajectory. The dive variability (dive_variability) in equation (4), is defined as the standard deviation of the raw profile. The average rate of change (dive_rate_of_change) in equation (5), is defined as the mean of the absolute differences between consecutive points in the profile (approximate first derivative). The number of “activity phases” (dive_activity_phases) in equation (6), is defined as the number of changes in the direction of the profile (approximate second derivative) detected by changes in the sign of the differences between consecutive values. These three features provide an indication of the extent to which the turtle has moved along the depth axis during its underwater activity. Lower values may indicate minimal movement, such as in resting phases, whereas higher values may indicate greater displacement, such as in chasing or escaping behaviours.

4 $$\,{\rm{dive\; variability}}=\sqrt{\frac{1}{n}\mathop{\sum }\limits_{i=1}^{n}{({{\rm{profile}}}_{i}-\mu )}^{2}}\quad \,{\rm{with}}\,\quad \mu =\frac{1}{n}\mathop{\sum }\limits_{i=1}^{n}{{\rm{profile}}}_{i}$$

5 $$\,{\rm{average\; rate\; of\; change}}\,=\frac{1}{n-1}\mathop{\sum }\limits_{i=1}^{n-1}\left|{{\rm{profile}}}_{i+1}-{{\rm{profile}}}_{i}\right|$$

6 $$\,{\rm{number\; of\; activity\; phases}}=\mathop{\sum }\limits_{i=2}^{n-1}| {\rm{sign}}({{\rm{profile}}}_{i+1}-{{\rm{profile}}}_{i})-{\rm{sign}}({{\rm{profile}}}_{i}-{{\rm{profile}}}_{i-1})| $$

#### Labelling the data

To facilitate measurement classification and to ease its interpretation by time of day or season, the previous dataset is enriched with text labels applied to features related to the dive and or surface events. Because the data spans several orders of magnitude for most of the features, we employed a logarithmic spacing that ensures that bins cover low and high values more evenly compared to linear binning. Features related to the duration and depth of dives have been grouped in five categories, ranging from “very short” to “very long” and from “very shallow” to “very deep”, respectively. Features related to surface event duration have been grouped in three categories named “short”, “medium” and “long”. All other features have also been grouped in three categories, named “low”, “medium” and “high”. In order to compute the bin range, the function first calculates the lower bound by taking the 1st percentile and upper bound by taking the maximum value. It ensures that the minimum value used for binning is not too small, which avoids issues if the column contains zeros or near-zero values that would interfere with logarithmic binning. We then start from these boundaries to create bins that are evenly spaced in logarithmic space The number of bins is determined by the number of labels provided. The Table 3 summarises the correspondence between concerned features, bins values and associated labels. Extreme values that lie beyond the bin ranges are assigned to either the first or last group, depending on whether they fall below or above the scale. Using the timestamp of each entry, we also assigned labels for the time of day and the current month, making it easier to interpret patterns based on daily or seasonal variations. To generate these labels, we used the Astral Python package, which is specialised in calculating various solar and lunar timings. The day is divided into five periods: “dawn” refers to the time from true dawn to thirty minutes after sunrise. Following this, the “morning” period lasts until the sun reaches its highest point (noon). The “afternoon” extends until thirty minutes before sunset, after which “dusk” covers the time until true dusk. The “night” period spans from dusk until dawn the following day.

**Table 2:** Detailed description of the features available in the initial dataset iot_all_turtles_preproc.csv.

Name	Description	Unit / Format
_time	record timestamp.	ISO8601
devEUI	tag 64-bit unique identifier.	hex string
battLevel_mV	tag battery level	mV
diveDeepHisto	array of five values representing the time spent inside a specific depth range. Ranges are [0.5,1,2,5] meters. Ranges for tags on the Reunion Island are different and equal to [3,10,15,20] meters.	sec
diveID	16-bit identifier of the dive event. This value is incremented each time the turtle starts diving.	integer
fcnt	16-bit mandatory LoRaWAN frame counter. This value is incremented each time the LoRa module sends a message.	integer
gatewayID	identifier for the receiving gateway. Correspondence between gateway ID, names and location is given in file data/gw_info_summary.csv.	integer
profile	raw dive profile represented as an array of 20 depth values with constant time steps specified in variable profile_tstep_s.	dm
rssi	LoRaWAN Received Signal Strength Indicator (RSSI). Traduces the received power signal.	dBm
snr	LoRaWAN Signal-to-Noise Ratio (SNR). The ratio between the received power signal and the noise floor power level.	dB
surfaceSensorUseTime	cumulative surface sensor use time. Corresponds to the total amount of time the turtle spent on the surface.	sec
temperature	average temperature recorded during the last dive event.	c°C
location	location where the turtle was captured	string
gatewayCnt	number LoRa gateway that have received the message if available. Equals to NaN otherwise	integer
tdive_s	duration of the last dive events	sec
profile_tstep_s	time steps of the last dive profile. This value is dynamically adjusted to adapt to longer dive duration	sec
maxdepth_m	maximum depth recorded in the last dive profile	m
avgdepth_m	average depth recorded of the last dive profile	m
profile_m	raw dive profile converted in SI unit	m
Name	tag name Correspondence between tag ID, name and turtle morphological measurements is given in file data/tag_info_summary.csv.	string
tsurf_s	time spent at surface before the last recorded dive events	sec

**Table 3:** Correspondence between concerned features, bins values and associated labels for the processed dataset iot_all_turtles_labelled.csv.

Feature	Description	Bins	Labels
tdive_s	duration of the last dive events (sec)	4 / 16.47 / 67.83 / 279.33 / 1150.30 / 4737	very-short / short / medium / long / very-long
avgdepth_m	average depth recorded of the last dive profile (m)	0.1 / 0.29 / 0.88 / 2.61 / 7.75 / 23.02	very-shallow / shallow / medium / deep / very-deep
maxdepth_m	max depth recorded of the last dive profile (m)	0.1 / 0.30 / 0.91 / 2.77 / 8.41 / 25.5	very-shallow / shallow / medium / deep / very-deep
tsurf_postdive_s	time spent at surface after the last recorded dive events (sec)	2 / 10.95 / 60.04 / 329	short / medium / long
tsurf_predive_s	time spent at surface before the last recorded dive events (sec)	2 / 10.95 / 60.04 / 329	short / medium / long
dive_activity_phases	number of changes in the direction of the profile (no unit)	2 / 4.23 / 8.97 / 19	low / medium / high
dive_rate_of_change	mean of the absolute differences between consecutive points in the profile (m/s)	0.01 / 0.07 / 0.52 / 3.88	low / medium / high
dive_variability	standard deviation of the profile (m)	0.02 / 0.17 / 1.43 / 11.91	low / medium / high
divetosurf_post_ratio	ratio between post-surface and dive duration (no unit)	0.12 / 3.27 / 86.83 / 2305	low / medium / high
divetosurf_pre_ratio	ratio between pre-surface and dive duration (no unit)	0.11 / 3.08 / 85.42 / 2368.5	low / medium / high

## Data Records

The datasets^22^ are stored and shared using the SEANOE platform, an open scientific data repository in the field of marine sciences. The repository is organised as follows:

- Folder data/ contains the four different versions of the dataset discussed above in CSV format. It also contains the detailed information about tagged individuals, their associated tags and information on the LoRa gateway deployed for the project.
  - File iot_all_turtles_preproc.csv: the initial version of the dataset with data for all tagged individuals across the 4 study sites.
  - File iot_all_turtles_filtered.csv: a cleaned version of the dataset where we removed about 2.3% of entries considered as outliers. The filtering steps have been explicated in the *Methods* section.
  - File iot_all_turtles_newfeatures.csv: the cleaned version but enriched with variables derived from the initial measurements of dive duration, surface duration and raw dive profile. The processing steps have been described in the *Methods* section.
  - File iot_all_turtles_labelled.csv: same version as above but with supplementary categorical variables to facilitate the data classification process. The labelling steps have been described in the *Methods* section.
  - File tag_info_summary.csv: a synthesis of the capture and tagging process with all details about the tag IDs and names, the capture and release times and locations, the identified species and the morphological parameters.
  - File gw_info_summary.csv: a synthesis of the deployed gateways with correspondence between IDs, names and precise locations.
- Folder photo_id/ contains 4 sub-folders with photos taken to identify tagged turtles. Individuals on Aldabra were not photographed.
- Folder output/ includes supplementary CSV files that contain information and results that have been calculated during the execution of the processing and analysis scripts.
- Folder figure/ contains PNG figures that have been generated during the execution of the processing and analysis scripts.
- At the root, we also included the Python scripts used to process and analyse the data, named data_analysis_X.py. They are supported by a README file that gives a brief description of their operation and usage. We also include a requirements.txt file to easily install all required packages in a Python environment.

Table 2 gives a detailed description of each column in the initial dataset, as in file iot_all_turtles_preproc.csv. New features, labels and associated bins added in the processed versions of the dataset are presented in Table 3.

The initial dataset has a total 100,875 records, where 54.6% of the data have been collected on the Europa Island, 27.5% on Mayotte, 12.6% on the Aldabra atoll and 5.3% on the Reunion Island. The median number of messages received per tag is 1371 (mean: 2967, min: 63, max: 14566). The details on data volume, transmission period and message frequency for each tag in operation is given in files output/nb_dive_and_msgloss_per_turtle.csv and output/iot_datavolume_timerange.csv. The filtering function described in section *Methods* has removed 2,233 records that were considered as outliers. Resulting in a total of 98,642 records for the three processed versions of the dataset.

## Technical Validation

### Hardware validation

Multiple tests were conducted to validate the robustness, functionality, and reliability of the tag hardware and embedded software under conditions representative of field deployments.

**Fig. 4:**
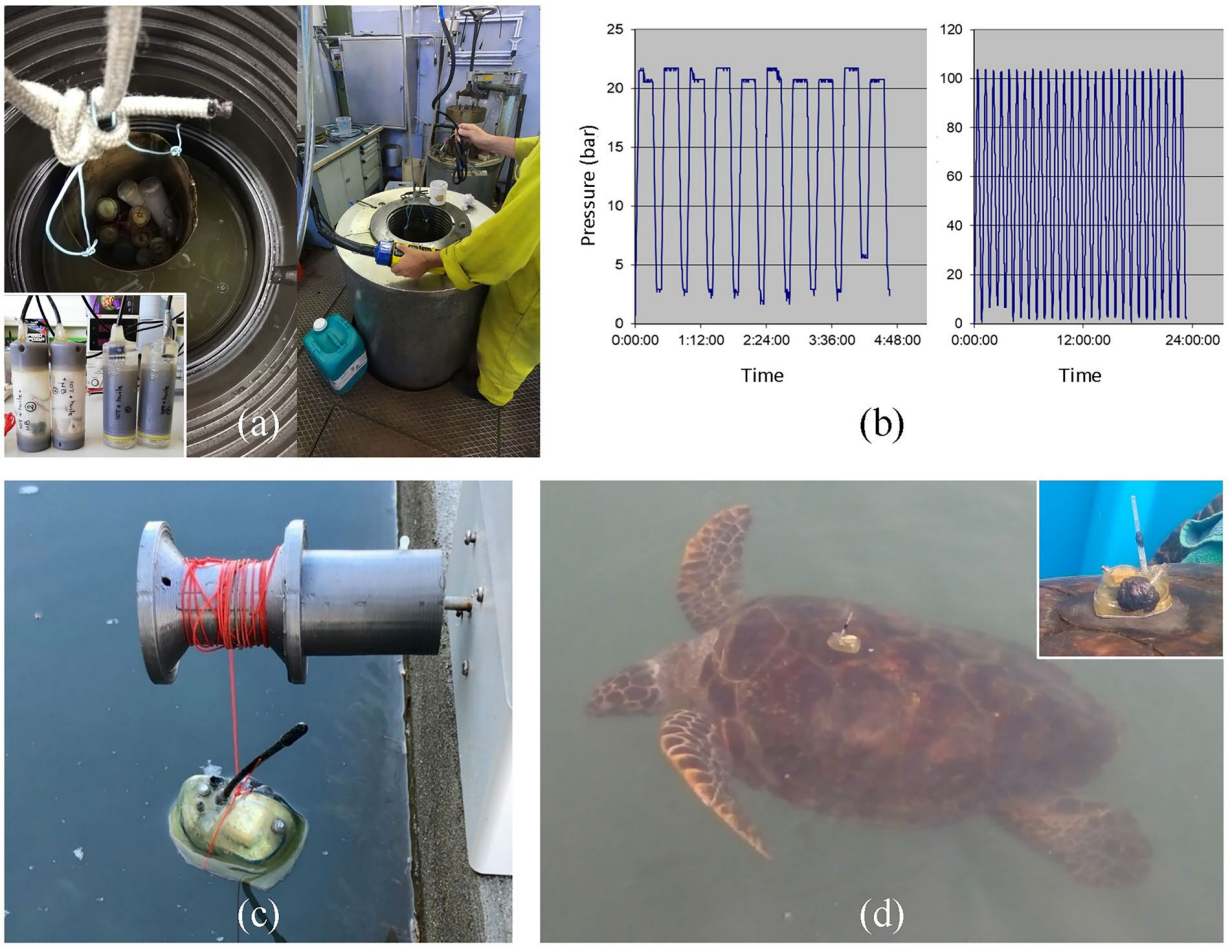
(**a**) Tag samples tested in the hyperbaric chamber at Ifremer’s laboratory in La Seyne-sur-Mer, operated by technical staff. (**b**) Pressure sensor output from one sample during hyperbaric testing: 10 cycles at 20 bar (left) and 28 cycles at 102 bar (right). (**c**) Automated test bench used to evaluate the robustness of the surface detection system. (**d**) Final integration test on a mature turtle in the main pond at the Kelonia Care Centre, Reunion. The tag shown is a prototype using the final electronics in an earlier casing design.

The electronic boards and external components of the tags—including the LoRa antenna and surface sensor electrodes—were tested in a hyperbaric chamber at Ifremer’s facility in La Seyne-sur-Mer, France, to assess resistance to pressure and waterproofing^38^ (Fig. 4a,b).

A series of tests was conducted to determine the optimal configuration for surface detection. These included experiments on electrode placement, pull-up resistance values, corrosion resistance, and waterproof coating durability. Automated bench tests using a motorized arm simulating dive/surface sequences confirmed the reliability of the pressure-based surfacing algorithm (Fig. 4c).

LoRa communication performance was evaluated both in lab and in open-water conditions^39^. Final tests at Kelonia Care Centre, Reunion Island, on captive turtles in a controlled environment confirmed proper tag operation over 12 days, including accurate surfacing and dive event detection (Fig. 4d). Battery life was identified as a constraint and addressed through the selection of a higher-capacity battery in the final tag version (Fig. 2).

### Comparison with literature data

In this section, we review previous studies on the dive and surface behaviour of juvenile sea turtles and compare them with our findings, highlighting consistencies and differences to assess how well our results align with existing research. Table 4 provides a summary of the relevant literature and compares it with the data from this study.

**Table 4:** Comparative analysis of juvenile sea turtle diving and surface behaviour across various studies.

Ref	Sites	Species	Dive depths (m)	Dive durations (min)	Surface events (sec)	Diel or seasonal pattern
^40^	Japan	Hawks.	3.7–16.6	10-70	23.6–42.8	Not specified
^41^	Florida, USA	Green	< 6	0.25–6	Short surface intervals, sometimes 1–2 sec	Not specified
^30^	Caribbean Coral Reefs	Hawks.	2–91	8–53	Not specified	Diel patterns: longer nocturnal dives
^44^	Coastal Waters, USA	Green	2–27	3.9 (avg)	Not specified	Depth and duration linked to turtle growth
^31^	Hawaii, USA	Green	Up to 4	3–9	< 15	Not specified
^42^	Caribbean Reefs	Hawks.	1.7–23.3	2.5–61.5	Not specified	Diel patterns: longer dives at night
^32^	Heron Island, Australia	Green	2.9–4.4	13–24	0.6–1.8	Longer, deeper dives in winter
^43^	Indian Ocean	Hawks.	0.2–2.4 (mean 0.8)	Diurnal: 7–22; Nocturnal: 17–61	0.1–0.5	Clear diel pattern: much longer nocturnal dives
This work	Indian Ocean	Green, Hawks.	25% < 0.3, 75% > 1.1	25% < 1.1, 75% > 11.2	25% < 3, 75% > 39	Detected diel patterns

Refs. ^40^: Okuyama *et al*.^40^, Salmon *et al*.^41^, Blumenthal *et al*.^30^, Hart *et al*.^44^, Francke *et al*.^42^, Witt *et al*.^31^, Southwood *et al*.

In our dataset, median dive durations range from 2.5 minutes at Europa Island to 6 minutes at Mayotte, with most dives lasting under 19.4 minutes. Surface events have median durations between 8 seconds (Mayotte) and 13 seconds (Réunion Island), with the majority under 60 seconds. Overall, 91.3% of dives have an average depth of less than 3 m. The most frequent depth category is “very shallow” ( < 0.3 m) at Aldabra and Europa, “medium” ( < 2.6 m) at Mayotte, and “deep” ( < 7.7 m) at Réunion. We note behavioral differences between sites, particularly in dive depth and duration, likely influenced by local habitat features (e.g. internal lagoons at Europa vs. outer reef slopes at Réunion) and site-specific foraging or predator conditions.

In the study^40^, the juvenile hawksbill turtles were observed to reach maximum dive depths of 16.6 meters, with mean depths during active dives ranging from 9.1 to 18.9 meters. Further, research conducted in^30^ revealed that juvenile hawksbills on Caribbean coral reefs exhibit dive depths ranging from 2 to 91 meters, with mean diurnal depths of 8 meters and nocturnal depths of 5 meters. In comparison, authors in^41^ reported that juvenile green turtles in the Florida current tend to stay in shallower waters, typically diving to depths less than 6 meters. Dive durations also show significant variation across studies, typically ranging between 1 and 44 minutes. In^40^, juvenile hawksbills in warmer waters were found to have average dive durations of 10 to 33.9 minutes for active dives, with resting dives extending from 30.3 to 70 minutes. Similarly, juvenile hawksbills in Caribbean coral reefs exhibited diurnal dives lasting an average of 16 minutes, while nocturnal dives tended to be longer, with a mean duration of 25 minutes^30^. Surface intervals between dives were found to be species- and context-dependent. In^40^, juvenile hawksbills exhibited post-dive surface durations ranging between 23.6 and 42.8 seconds. In coastal habitats, juvenile green turtles were reported to have brief surface intervals, often lasting less than 15 seconds^31^. Additionally, in^41^, green turtles were sometimes observed surfacing for as little as 1-2 seconds before diving again. Studies on juvenile hawksbills, such as those in^8^ for the Indian Ocean and^42^ for the Caribbean, did not provide detailed surface interval data, though frequent surfacing was reported, particularly during shorter foraging dives. Seasonality has also been shown to significantly influence diving behaviour in some species. In colder seasons, juvenile green turtles in subtropical waters of Australia exhibited longer dive durations, averaging 24.3 minutes, compared to 13.1 minutes in the summer^32^. Authors in^30^ and^42^ further reported that juvenile hawksbills display pronounced diel patterns, with longer and deeper dives occurring at night compared to daytime dives. However, studies such as^31^, which focused on juvenile green turtles in coastal neritic habitats, did not report explicit seasonal variations, attributing diving behaviour more to habitat characteristics. Recent work in the Chagos Archipelago, also located in the Indian Ocean, further highlights the diversity of shallow-water diving strategies in juvenile hawksbills. Authors in^43^ reported extremely shallow dives (<3 m) with mean bottom depths around 0.8 m and notably long durations, particularly at night (17-61 min) despite warm water temperatures (24-37 °C). Surface intervals were remarkably short (0.1-0.5 min), and diel patterns were pronounced.

## Data Availability

All data and analysis scripts associated with the current submission have been included in the SEANOE repository (https://www.seanoe.org/data/00914/102544/) or are also available on the dedicated GitHub project (https://github.com/ocean-monitoring-gateway/dataset-seaturtle-swio-2021). The dataset^22^ is published under the CC BY 4.0 licence. We request that users of these data cite this manuscript in any publication that results from its use. The authors are available for consultations about and collaborations involving the data. Instructions on how to install and run the scripts, as well as a short description of what they do, are given in the GitHub project’s README file. Using the code only requires basic programming skills in Python and familiarity with CSV file manipulation for data exploitation and visualization.
